# Exploring nutrition-related interests of the transgender and gender-diverse community: a content analysis of a transgender-centric discussion forum on Reddit

**DOI:** 10.1017/S1368980024001459

**Published:** 2024-09-23

**Authors:** Heather E Schier, Krithika Chetty, Shivakriti Induri, Julie Kennel, Carolyn Gunther

**Affiliations:** 1 Department of Nutrition and Healthcare Management, Appalachian State University, Boone, USA; 2 Department of Human Sciences, The Ohio State University, Columbus, USA; 3 College of Public Health, The Ohio State University, Columbus, USA; 4 College of Arts and Sciences, The Ohio State University, Columbus, USA; 5 College of Nursing, The Ohio State University, Columbus, USA

**Keywords:** Transgender nutrition, Information-seeking behaviours, Social media health advice, Hormone replacement therapy

## Abstract

**Objective:**

The solicitation of nutrition-related health advice on social media platforms is on the rise. However, there is a paucity of research on the distinctive nutrition-related concerns and needs faced by transgender and gender-diverse individuals. Understanding patterns of nutrition-related information-seeking behaviour is vital to advancing health promotion efforts within this community. This study aimed to characterise the nutrition-related questions posed by the transgender community on a prominent social media outlet, Reddit.

**Design:**

A qualitative, cross-sectional content analysis was conducted, focusing on the top 100 submissions (ranked by popularity) within a transgender-centric online subreddit (r/asktransgender). Data extraction was facilitated using the Application Programming Interface Pushshift. The content analysis was conducted using NVivo.

**Setting:**

The study was situated within the discussion forum of the social media platform, Reddit.

**Results:**

A total of 148 references from 90 eligible posts were identified and coded. The major themes included the effects of hormone replacement therapy (HRT) on nutritional health (*n* 66), weight status (*n* 45), dietary needs and behaviours (*n* 21), physical activity and weight loss on body shape (*n* 9), social undermining (*n* 4) and effects of health behaviours on HRT (*n* 3).

**Conclusion:**

This study underscores the pressing need for tailored and evidence-based nutrition guidelines and communication toolkits that specifically address the distinct needs and experiences of transgender individuals, particularly those undergoing HRT.

Increasingly, people are turning to the web for answers to health-related questions. This trend is particularly prevalent among underrepresented communities, like the transgender and gender-diverse (TGD) population, that experience barriers to equitable healthcare access^(1)^. A survey of over 900 TGD individuals reported the Internet is their preferred source of health-related information, citing Reddit as the most popular online platform^(2)^. Preliminary data demonstrate similar patterns among TGD youth (13–24 years of age), who shared that the Internet was a primary source of health and nutrition-related information.

TGD individuals across life stages appear to disproportionately experience nutrition-related health disparities such as disordered eating and food insecurity^(3–6)^. Disparities may arise from limited access to culturally competent healthcare, hormone replacement therapy (HRT) effects on metabolism and appetite, mental health factors impacting eating habits and societal discrimination influencing nutritional choices (e.g. barriers to accessing food assistance programmes, influence of weight and body shape bias on eating patterns, social norms that genderize foods, etc.), potentially leading to higher risks of nutritional deficiencies or imbalances^(3–6)^. Given the unique nutrition-related concerns experienced by TGD individuals, understanding the patterns of nutrition-related information-seeking behaviour is critical to promoting health. However, current nutrition guidelines are sex-specific, making it difficult for TGD individuals to apply them to their own experiences, particularly those undergoing HRT^(7,8)^. A growing body of literature is exploring the nutrition-related concerns and interests of the TGD community. For example, a qualitative analysis of the nutrition-related messages shared by transgender vloggers on YouTube identified distinct nutrition concerns and interests related to the transgender experience, such as body composition shifts as a result of HRT^(9)^.

As researchers delve into the nutrition-related concerns of TGD individuals, the exploration of information-seeking behaviour becomes crucial. Reddit is a popular social news aggregation and discussion platform, known for its vast array of communities, or subreddits, dedicated to specific topics and interests^(10)^ and has become a space of exploration for researchers interested in understanding the content and trends of information sharing. Among health-related topics, nutrition and diet are among the most frequently sought information on Reddit^(11)^.

Reddit’s user demographics vary across subreddits and topics. Generally, according to a Pew Research Center survey from 2019, Reddit is more popular among men than women, with a strong presence among young adults^(12)^. However, it is essential to recognise that the user base may have evolved since the survey, and demographics may differ among specific subreddits, including those centred around transgender topics.

Transgender individuals find Reddit to be a valuable, anonymous platform for seeking information and resources^(1,13)^. The r/asktransgender subreddit, alongside other related communities, fosters open discussions and encourages users to ask questions, share personal stories and seek advice from others who may have similar experiences. This virtual space has become a popular resource for those looking to navigate various aspects of transgender life, including health concerns^(1,13)^. However, few studies have explored the nature of health-related information shared, particularly nutrition-related information among transgender-centric subreddits. Therefore, this content analysis aimed to characterise the nutrition-related questions posted on a transgender-centric discussion forum (subreddit) on Reddit to gain a deeper understanding of the nutrition-related interests of the TGD community. These findings are expected to add nuance to the growing body of literature that is uncovering the nutrition-related priorities and concerns of the TGD community.

## Methods

### Qualitative approach and paradigm

Content analysis is a systematic method for analysing and interpreting text data^(13)^. This method identifies themes and patterns within the text. In the context of Reddit posts, content analysis allows researchers to explore and understand the nature of discussions, identify prevalent themes and gain insights into user perspectives. A constructivist paradigm underpinned the study design and execution. Constructivism emphasises the subjective nature of reality and recognises that knowledge is socially constructed^(14)^. In the case of analysing Reddit posts, constructivism is appropriate because it acknowledges that the meanings and interpretations of the content are influenced by the participants’ perspectives, interactions and the context of the online community.

### Data extraction

Pushshift is an Application Programming Interface (API) that provides access to Reddit’s entire history of posts and comments, going back to 2005. It allows users to search and retrieve posts and comments from Reddit’s public API that have been archived on the Pushshift servers. The Pushshift API is useful to analyse large amounts of Reddit data without running into the rate limit restrictions of Reddit’s public API. To use the Pushshift API, users send HTTP requests to the API endpoint with parameters that specify the search criteria, such as keywords, subreddit, date range and other filters. The API returns JSON-formatted data that includes metadata, descriptive information about data and content for each post or comment that matches the search criteria.

For this study, Pushshift API was used to conduct a search of relevant content. The following keywords were searched across the top transgender-centric subreddit used in other related studies, r/asktransgender^(15)^: diet, nutrition, consume, eat, appetite, food, drink, weight, soy, protein, carbohydrate, lipid, fat, vitamin, supplement, energies, macro and hunger (Appendix A). Keywords were selected based on relevance to nutrition and dietary behaviours. Experts (2 registered dietitians) were consulted, and revisions were made accordingly. We tested different search strings in preliminary searches, ultimately selecting the set of keywords that yielded the highest number of relevant results. Results were limited to the top 100 submissions, sorted by popularity, based on number of interactions (i.e. upvotes). A systematic review of similar studies that used Reddit data revealed over 50 studies have employed sample frames with fewer than 100 posts. Given the exploratory nature of this study focused on a specific niche topic, the top 100 posts provide valuable insights to guide future research in meaningful directions^(16,17)^. Parameters were selected to ensure the data extracted represented timely, focused and salient content. The results were converted from a JSON file to a legible tree format and uploaded to NVivo 12 for analysis.

### Data analysis

A content analysis of the top 100 nutrition-related questions posted on the subreddit, r/asktransgender, was conducted in NVivo 12. Independent coders (*n* 3) conducted open line-by-line coding of the first 10 % of the material, assigning labels (‘nodes’) to each relevant unit of text. Nodes were developed organically, without a predefined codebook. The data segments were not predetermined; instead, they were coded *ad hoc* based on what the coders perceived as conceptually significant. The coding process allowed for multiple nodes to be assigned to a single data segment, enabling a more comprehensive analysis. Coders met to discuss node selection and reconciled differences. Two coders continued to code independently until intercoder agreement of > 0·9 was reached. At that time, one coder analysed the remaining content. NVivo 12 software was used for the next phase, axial coding, a process of identifying trends and patterns in topics across nodes. One coder reviewed and categorised nodes into central themes through an iterative process balancing inductive reasoning (themes identified organically through data) and deductive reasoning (relating content to existing theories and concepts relevant to the research question). This resulted in the generation of a codebook organised into themes and subthemes. Parent nodes were aggregated by child nodes. Frequencies of themes and subthemes were calculated.

### Reflexivity statement

The content analysis was conducted by three cisgender females, two falling within the same age group as the target population. The team comprised individuals with diverse backgrounds – one identifying as white/Caucasian and the other two as Brown/Asian Indian. Notably, one researcher brings over 5 years of research experience with transgender communities.

Together, the team amalgamates expertise in community nutrition, behaviour change theory and public health. Throughout the research process, the team conscientiously reflected on their own backgrounds and positionalities, recognising the potential influence on various facets of the study, including the interpretation of subjective knowledge. It is imperative to emphasise that the nuanced interpretation of subjective findings is intricately connected to the unique perspectives and identities of the researchers involved in this study.

## Findings

**Fig. 1:**
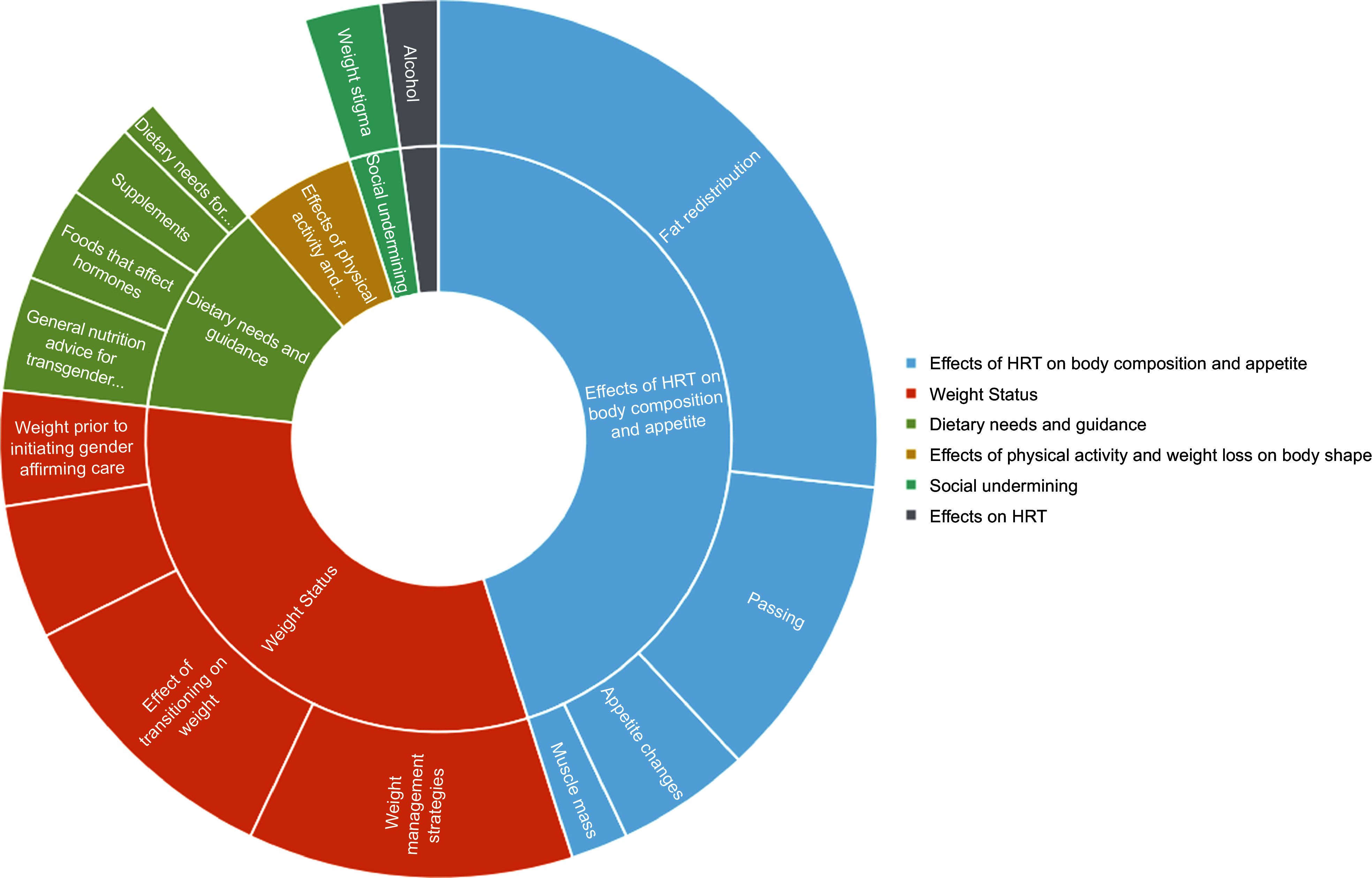
Sunburst chart with distributions of nutrition-related themes and subthemes of questions submitted to a transgender-centric Reddit forum

The search resulted in 90 relevant original posts from 12/13/22 to 3/16/23. Ten posts were excluded because the content was not related to nutrition or a question was not asked. 148 references across the ninety posts were identified and coded. The major themes (Table 1 and Fig. 1) included the effects of HRT on nutritional health (*n* 66), weight status (*n* 45), dietary needs and behaviours (*n* 21), physical activity and weight loss on body shape (*n* 9), social undermining (i.e. the intentional or unintentional actions that weaken or sabotage another person’s social standing, relationships or well-being) (*n* 4), effects of health behaviours on HRT (*n* 3). Table 1 outlines the themes and subthemes identified, frequency across the data set and representative quotes.

**Table 1:** Six major themes and subthemes of the nutrition-related questions submitted on a transgender-centric subreddit on Reddit

Themes	Subthemes	Frequency		Representative quotes
		*n*	%	
Effects of HRT* on body composition and appetite	Fat redistribution	38	25·5	‘Does initial body weight effect the fat redistribution effect of GAHT? Say one is an overweight individual, is it advisable to try and start from a low body fat state for best results or does it not matter?’
	Passing†	16	10·7	‘How does one deal when passing stops after weight loss? So my current understanding of how HRT effects fat is that it’ll change where new fat is stored but not change the fat I already have. With that in mind I’ve been losing a lot of weight so far, started at 283 in September and am down to 229 now. (I’m 6’3) I plan on going down to like 160 with which I’ll probably be too skinny for my liking so that I can lose as much male placed fat as I can then gain back up to like 180–185. I’m hoping this will give me a much more feminine figure than I currently have. Does this make sense? Has anyone else done something like this, how did it work for you? Thanks!’
	Muscle mass	3	2·0	‘How to achieve Weight loss without gaining masc appearance? AMAB Pre-everything.’
	Appetite changes	7	4·7	‘Is a decrease in appetite normal on mtf HRT?’
Weight Status	Weight management strategies	17	11·0	‘How are trans women able to lose and gain so much weight during weight cycling?’
	Effect of transitioning on weight	15	10·0	‘Is weight loss more difficult on hormones?’
	Weight and passing†	7	4·7	‘I have been on hrt for 23 months as of February. I have a really little breast growth even though my T has been adequately suppressed since last year. I am barely an A cup now. I have read that being under weight can give poor progress and physical changes on HRT. I have a low body, due to poor eating habits and lifestyle choices. Now I am planning to gain weight but I am afraid of male pattern fat distribution. If anyone went through the same experience and has any advice then please suggest.’
	Weight prior to initiating gender-affirming care	6	4	‘Is there a minimum weight for [HRT]? Mine is 47 kg, I measure 165 cm. So I’m below ideal weight (I’d need 3 more kg), is that something that would stop doctors?
Dietary needs and guidance	General nutrition advice for transgender individuals	6	4	‘Whenever I look up any food ingredient, I see “men’s health” and “women’s health.” I was thinking: if a trans person on hrt wanted nutrition tips, what would they follow?’
	Foods that affect hormones	5	3·4	‘What are some healthy foods I can eat before hormone blockers that either boost oestrogen or decrease testosterone?’
	Supplements	4	2·7	‘Are there any vitamins I can get that can help increase my oestrogen levels. I haven’t started hormones yet and it’s probably going to be awhile before I can.’
	Dietary needs for transgender and gender-diverse individuals	2	1·3	‘Being trans, how much fibre should I eat per day? The question is easy enough to search online for cis people, and the answers I’ll see often recommend that men eat 30–38 g/d while women eat 21–25 g/d. Since I am MTF and on HRT, which of these should I follow? I’ve been generally aiming for 21–25, but I’ve always felt unsure.’
Effects of physical activity and weight loss on body shape		9	6	‘FTM here, does working out and losing weight make your chest smaller or more prominent?’
				‘Heya, I used to be a water polo player so I have some pretty wide shoulders and big arms, but I am generally considered skinny so my arms looks very out of place…I know that I have to be eating in a energy surplus, but is there any way I can also work out to slim down my arms and maybe my waist without losing the gains on my glutes?’
Social undermining	Weight stigma	4	2·7	‘When a cisgender person compares you being trans to them being fat… Why the fuck do they do this? What do they think trans is…How does having missing body parts even compare to being fat? Please help my fat trans ass understand!’
Effects on HRT*	Alcohol	3	2	‘Is it bad for me to drink on HRT?’
Total References		148		

* GAHT = gender-affirming hormone therapy, HRT = hormone replacement therapy, AMAB = assigned male at birth; MTF = male-to-female; FTM = female-to-male.

† Passing, colloquial term to describe the experience of being correctly gendered by others, often described as an alignment between one’s physical appearance and gender.

### Effect of hormone replacement therapy on body composition and appetite

The data in this study revealed that the effect of HRT on body composition and appetite was of interest. Notably, fat redistribution was a prominent concern among users, indicated by the primary code ‘fat redistribution’ with thirty-eight occurrences. Within this code, two subcodes emerged: ‘muscle mass’ (*n* 3) and ‘passing’, or the ability to be recognised as one’s gender (*n* 16). This nuanced theme is exemplified by a participant’s inquiry,

*‘Does initial body weight effect the fat redistribution effect of GAHT (Gender-Affirming Hormone Therapy)? Say one is an overweight individual, is it advisable to try and start from a low body fat state for best results, or does it not matter?’*

This inquiry exemplifies the multifaceted nature of user concerns and underscores the perceived complex impact of HRT on body composition and vice versa.

### Weight status

Weight-related inquiries (*n* 45) emerged as a prominent and recurrent theme, often intersecting with questions about fat redistribution. Subthemes included weight management strategies (*n* 17), effect of transitioning on weight (*n* 15), weight changes and passing (*n* 7) and weight prior to initiating gender-affirming care (*n* 6). These inquiries spanned a spectrum, from concerns about the potential influence of being underweight on the effectiveness of HRT to seeking guidance on managing weight while aligning with their gender expression goals, such as ‘passing’ as their affirmed gender. Weight cycling emerged as a recurring topic, exemplified by a user’s query:

*‘How are trans women able to lose and gain so much weight during weight cycling?’*

Additionally, some individuals articulated a concomitant desire to change body weight (e.g. weight gain) to optimise health-related goals and fear of adverse consequences on gender-affirming physical attributes. For instance, a user shared,

*‘I have been on hrt for 23 months as of February. I have a really little breast growth even though my T has been adequately suppressed since last year. I am barely an A cup now. I have read that being underweight can give poor progress and physical changes on HRT. I have a low body, due to poor eating habits and lifestyle choices. Now I am planning to gain weight but I am afraid of male pattern fat distribution. If anyone went through the same experience and has any advice then please suggest’*

This illustrates the complex dynamics often encountered during gender-affirming journeys, particularly the intricate interplay between weight, physical transformations and HRT on one’s gender-affirming characteristics.

### Dietary needs and behaviours

There was heterogeneity among the topics of interest within this overarching theme. The topics included dietary needs for transgender individuals, supplement recommendations for feminising or masculinising (*n* 4), general nutrition advice for transgender persons (*n* 6), food and diet that influence hormone levels (*n* 5) and dietary recommendations for those on HRT and Spiro [specifically seeking fibre recommendations and inquiring if they should be concerned about potassium deficiencies while on spironolactone (a potassium-sparing diuretic)] (*n* 2). An illustrative query reflecting foods that influence hormone levels was,

*‘What are some healthy foods I can eat before hormone blockers that either boost oestrogen or decrease testosterone?’*

Some posts highlighted the recognition that nutrition guidelines often carry a sex-specific orientation, prompting users to seek advice on adapting these guidelines to align with their gender identity and medical transition status. A user expressed this sentiment, stating,

*‘Whenever I look up any food ingredient, I see “men’s health” and “women’s health.” I was thinking: if a trans person on HRT wanted nutrition tips, what would they follow?’*

Conversely, certain questions transcended gender specificity, such as those focused on regulating bowel movements and soliciting general meal-planning advice. This diversity underscores the multifaceted nature of dietary considerations among the transgender community and their unique intersections with gender identity, medical transition and general well-being.

### Effects of physical activity and weight loss on body shape

This theme encompassed inquiries (*n* 9) related to utilising exercise and/or weight loss strategies to achieve desired body shape goals, often aligned with their gender expression objectives. Participants commonly discussed topics such as targeting specific muscle groups through resistance training and diet modification. They sought guidance on various aspects, including the potential impact of exercise and weight loss on natural fat redistribution during HRT, exercise and dietary recommendations prior to initiating HRT, reducing chest size and methods to target muscle groups for controlling body shape. For instance, one user expressed concerns about developing a curvier figure through resistance training, particularly focusing on glute workouts, and maintaining a positive energy balance in their diet, without inadvertently bulking up and acquiring more defined muscles typically associated with masculinity. Another area of interest revolved around achieving weight loss while avoiding fat distribution in the chest and hips.

*‘FTM [Female-to-male] here, does working out and losing weight make your chest smaller, or more prominent?’*

Noteworthy topics identified but infrequently: social undermining, specifically weight bias (*n* 4), concerns about whether alcohol intake would negatively affect HRT (*n* 3), food habits (*n* 1), impact of HRT on blood pressure (*n* 1), effect of undernutrition on HRT effectiveness (*n* 1), posing a generalising question with intent to offer advice (*n* 1) and coping with discrimination by a food service worker (*n* 1).

## Discussion

This study aimed to identify prevalent nutrition-related topics of interest to the transgender community by examining queries on a Reddit subreddit (r/asktransgender). With the increasing reliance on the Internet for health-related information, it is crucial to understand the patterns of information-seeking behaviour among underrepresented communities, such as the TGD population^(1)^. TGD individuals often face barriers to equitable healthcare access, and the Internet has become their preferred source of health-related information^(1)^.

Findings from the present study are expected to provide nuanced insights into the nutrition-related information sought by TGD individuals and inform the development of tailored and evidence-based nutrition guidelines that address their specific needs and experiences.

While no prior investigation has specifically examined nutrition-related inquiries on a transgender-focused Reddit forum, a netnographic cross-sectional study analysed information disseminated by transgender YouTube vloggers. This study identified analogous themes to the current investigation, encompassing inquiries about the effects of HRT on weight and body fat redistribution, dietary needs as a TGD individual and across gender-affirming care services, general dietary quality, dietary supplements and the effects of physical activity on body shape^(9)^.

One prominent theme identified in the present study was the effect of HRT on fat redistribution, with specific subcodes related to muscle mass and passing. These results underscore the interest of individuals undergoing HRT in understanding its direct effects on body composition, particularly in terms of fat redistribution and muscle development. The desire to achieve a physical appearance aligned with their gender identity and the ability to pass as their gender were recurring themes within this subtheme. Notably, a major concern lies at the intersection between weight loss, fat redistribution and repressed ‘passing’, leaving individuals feeling more dysphoric as some sex-based physical features become more pronounced after weight loss (e.g. feminine hip structures and masculine muscle distributions). Additionally, questions about appetite changes in response to HRT were raised by seven users, indicating a need to explore the potential impact of HRT on eating behaviours.

Weight status was identified as another significant theme, with users seeking information about the implications of being underweight on HRT effectiveness and advice on weight loss while maintaining their desired gender expression goals, such as passing as their gender. Notably, TGD individuals disproportionately experience weight concerns compared to their cisgender counterparts, aligning with previous literature^(6,18,19)^.

Within the overarching theme of dietary needs and behaviours, a diverse range of topics were discussed. These included specific dietary needs for transgender individuals, supplement recommendations for feminising or masculinising effects, general nutrition advice for transgender persons, the influence of food and diet on hormone levels and dietary recommendations for those on HRT and Spironolactone. Some users sought guidance on adapting sex-specific nutrition guidelines to their gender and medical transition status. Others inquired about regulating bowel movements and seeking meal-planning advice. Dietary supplements have been indicated as an area of interest among the TGD community in other work^(9,19,20)^. One study investigated the prevalence and associations of appearance and performance-enhancing drugs and supplements use among gender minority individuals, revealing that a significant proportion, including 30·7 % of gender-expansive people, 45·2 % of transgender men and 14·9 % of transgender women, reported lifetime appearance and performance-enhancing drugs and supplements use, with notable correlations with eating disorder and muscle dysmorphia symptoms in gender-expansive people and transgender men, emphasising the need for clinical assessment of these behaviours in gender minority populations^(19)^. Further, a cross-sectional survey found significantly higher rates of dietary supplement use among transmasculine individuals compared to the general public, with 64·5 % reporting recent and 90·0 % lifetime use of dietary supplements^(20)^.

The theme of physical activity and weight loss centred around users’ desire to achieve specific body shape goals in line with their gender expression. Users sought guidance on targeting muscle groups through resistance training, managing weight loss while preserving desired fat distribution and reducing chest size for trans masculine/androgenous individuals or controlling body shape for trans feminine individuals. This theme aligns with other literature^(9,21)^. In a needs assessment study conducted at a midwestern university, approximately 50 % of the TGD participants reported trying to lose weight and 88 % changed their eating patterns to change their body^(21)^. These inquiries shed light on the intricate relationship between exercise, weight loss and body shape goals among transgender individuals, necessitating further investigation and guidance to address their specific concerns and preferences.

Topics such as the effects of alcohol intake on HRT, food habits, impact of HRT on blood pressure and coping with discrimination by a food service worker were mentioned but to a lesser extent. It is worth noting that although inquiries regarding weight stigma were not explicitly posed, it is conceivable that this crucial issue^(21)^ may be implicitly interwoven within other Reddit submissions. For instance, it could manifest in narratives shared across various forums rather than being explicitly directed to the ‘r/asktransgender’ community. Recognising the multifaceted nature of these online discussions, future exploration of these themes could contribute to a more comprehensive understanding of the diverse nutrition-related challenges faced by individuals undergoing HRT and navigating TGD experiences.

Overall, these findings highlight the complex relationship between HRT, nutritional health, weight management and body shape goals for TGD individuals. They underscore the need for further research and guidance to address the specific concerns and preferences within the transgender community, including tailored nutritional recommendations, strategies for managing appetite changes and addressing weight stigma in healthcare settings.

### Strengths and limitations

The study has several strengths in its methodology that contribute to its robustness. Firstly, the utilisation of the Pushshift API to extract data from Reddit ensures access to a vast amount of publicly available content without being constrained by rate limit restrictions. Additionally, the specific search criteria and keywords employed in the study were well-defined, focusing on relevant nutrition-related terms within the targeted subreddit, and were reviewed by topic experts. By sorting the results by popularity, the study captures the most widely discussed and engaged-with content. The use of NVivo 12 for data analysis further enhances the rigour of the study, allowing for systematic content analysis and identification of themes and patterns. The involvement of multiple independent coders and intercoder agreement checks ensures the reliability and consistency of the coding process. Additionally, capturing engagement on an anonymous social media site offered more diverse and rich information and perspectives. Finally, to our knowledge, no other investigator has characterised the nutrition-related questions submitted on Reddit among the transgender community.

This study also has limitations. First, submissions analysed were limited to the top 100 posts on the r/asktransgender subreddit, which may not representatively capture the nutrition and diet-related questions posted on the platform. It is possible that there are frequent posts on these topics beyond the scope of this study. Secondly, as the study focused on a specific timeframe, it does not provide insights into any potential changes or trends in the nutrition-related questions over time. Future studies could consider capturing the top 100 posts each year over recent years to compare and analyse any divergences or evolving patterns in the questions posed by the community. Doing so would provide a more comprehensive understanding of the nutrition-related interests and concerns of the transgender community on Reddit.

### Future research directions

Several potential future research directions can extend the inquiry in this field. Firstly, investigating differences in the topics queried by geographic location could provide valuable insights into the cultural and regional nuances of nutrition-related concerns within the transgender community. This could include exploring differences by state-level transgender legislature. Additionally, conducting similar studies in related subreddit forums dedicated to transgender health and well-being could offer a broader perspective and allow for comparisons across different online communities. Exploring additional nutrition-related topics beyond those covered in this study, such as specific dietary interventions, micronutrient needs or the impact of nutrition on HRT outcomes, would provide a more comprehensive understanding of the unique nutritional needs and challenges faced by transgender individuals. These future research directions can contribute to a more nuanced and evidence-based approach to addressing the nutrition-related concerns of the transgender community and promoting their overall health and well-being.

## Conclusion

This study shed light on the unique nutrition-related concerns and interests of the TGD community. The analysis underscored key concerns, including fat redistribution, strategies for weight management and the intricate interplay between HRT-induced weight alterations and the pursuit of gender expression goals. These findings emphasise the imperative for the development of precise, evidence-based nutrition guidelines tailored to the unique needs and experiences of transgender individuals, particularly those in the process of HRT.

## Appendix A. Search strategy for API pushshift to return nutrition-related questions submitted on the subreddit, r/asktransgender

List of Keywords Used in Reddit Search

String: with OR operators: diet|nutrition|consume|eat|appetite|food|drink|weight|soy|protein|carbohydrate|lipids|fat|vitamins|supplements|calories|macros|hunger

diet nutrition consume eat appetite food drink weight soy protein carbohydrate lipids fat vitamins supplements calories macros hunger

Parameters:

Search submissions only (not comments)

Subreddit = r/asktransgender

# of submissions returned = 100

Sort by popularity in descending order

Include date created, author flair

API Pushshift Requests

Attempt 1:

https://api.pushshift.io/reddit/search/submission/?subreddit=asktransgender&size=100&sort_type=score&score=desc&filters=score,size,created_utc,total_results&q=diet|nutrition|consume|eat|appetite|food|drink|weight|soy|protein|carbohydrate|lipids|fat|vitamins|supplements|calories|macros|hunger

https://api.pushshift.io/reddit/search/submission/

?subreddit=asktransgender

&size=200

&sort_type=score

&sort=desc

&fields=created_utc,author_flair_text,author_flair_css_class

&q=diet|nutrition|consume|eat|appetite|food|drink|weight|soy|protein|carbohydrate|lipids|fat|vitamins|supplements|calories|macros|hunger

#############################################

Attempt 2 (FINAL):

https://api.pushshift.io/reddit/search/submission/?subreddit=asktransgender&size=100&fields=selftext,score,created_utc,total_results&title=(diet|nutrition|consume|eat|appetite|food|drink|weight|soy|protein|carbohydrate|lipids|fat|vitamins|supplements|calories|macros|hunger)

This request returned up to 100 submissions from the subreddit r/asktransgender that contain any of the terms in the search query. The results (should) include only the following fields:

selftext: The full text of the submission.

score: The net score of the submission (upvotes minus downvotes).

created_utc: The timestamp of the submission, in UTC.

total_results: The total number of results that match the search query.

Note that the fields parameter is used to specify which fields should be included in the results, and the title parameter is used to search for the desired terms in the title of the submissions. If you wanted to include additional fields, you could simply add them to the fields parameter separated by commas.
